# Recent advances in understanding the regulation of plant secondary metabolite biosynthesis by ethylene-mediated pathways

**DOI:** 10.1007/s12298-024-01441-w

**Published:** 2024-03-31

**Authors:** Alka Tripathi, Nisha Chauhan, Pradipto Mukhopadhyay

**Affiliations:** 1 Plant Biotechnology division, CSIR-Central Institute of Medicinal and Aromatic Plants, Lucknow, Uttar Pradesh 226015 India; 2 Academy of Scientific and Innovative Research (AcSIR), CSIR-HRDC Campus, Ghaziabad, Uttar Pradesh 201002 India

**Keywords:** Ethylene signaling, Secondary metabolites, MVA/MEP Pathway, Phenylpropanoid pathway, Regulation and biosynthesis

## Abstract

Plants produce a large repertoire of secondary metabolites. The pathways that lead to the biosynthesis of these metabolites are majorly conserved in the plant kingdom. However, a significant portion of these metabolites are specific to certain groups or species due to variations in the downstream pathways and evolution of the enzymes. These metabolites show spatiotemporal variation in their accumulation and are of great importance to plants due to their role in development, stress response and survival. A large number of these metabolites are in huge industrial demand due to their potential use as therapeutics, aromatics and more. Ethylene, as a plant hormone is long known, and its biosynthetic process, signaling mechanism and effects on development and response pathways have been characterized in many plants. Through exogenous treatments, ethylene and its inhibitors have been used to manipulate the production of various secondary metabolites. However, the research done on a limited number of plants in the last few years has only started to uncover the mechanisms through which ethylene regulates the accumulation of these metabolites. Often in association with other hormones, ethylene participates in fine-tuning the biosynthesis of the secondary metabolites, and brings specificity in the regulation depending on the plant, organ, tissue type and the prevailing conditions. This review summarizes the related studies, interprets the outcomes, and identifies the gaps that will help to breed better varieties of the related crops and produce high-value secondary metabolites for human benefits.

## Introduction

Plants synthesize various classes of secondary metabolites (SMs), such as alkaloids, flavonoids, saponins, lactones, terpenes, steroids, etc. that are known to confer specific odour, taste, and colour to plants and play a key role in the plant’s growth and development, interaction with the environment, and defence against biotic stresses (Erb and Kliebenstein 2020). More than 2,140,000 SMs are known, of which 200,000 to 1,000,000 specialized metabolites have been estimated to be produced by plant kingdom (Rai et al. 2017; Pant et al. 2021). Many of these metabolites, especially from medicinal and aromatic plants (MAPs), are significant for their utility as therapeutics, nutraceuticals, base compounds for drug development, and aromatics in culinary and cosmetics preparations. Phenolics, terpenoids, and alkaloids, the three major broad classes of plant SMs, are biosynthesized by four major biochemical pathways, *viz*. the malonic acid pathway, the shikimate pathway, the methyl erythritol phosphate (MEP) pathway and the mevalonic acid (MVA) pathway.

Ethylene is the simplest known gaseous plant hormone, which classically is known for its activities related to ripening of climacteric and more recently, non-climacteric fruits (Fugate et al. 2010; Ustun et al. 2023). In addition, ethylene also has a major role in generating triple response in plants producing climacteric fruits which includes inhibition of the root and hypocotyl elongation, exaggerated tightening of the apical hook, and swelling of the hypocotyls (Chen et al. 2018). Interestingly, a few plants, like melons and pears, have varieties which differ by producing fruits of either climacteric or non-climacteric nature (Saladié et al. 2015). In rice, ethylene influences seed dormancy and germination (Frenkel et al. 2020) and stimulates root initiation and development (Song et al. 2016). Contrarily, it inhibits storage root formation in plants like *Rehmannia glutinosa,* and represses lateral root initiation in tomatoes (Negi et al. 2010; Wang et al. 2014). The role of ethylene in providing resistance against wound and biotic stress is well-studied (Natalini and Palma 2023). However, it also manifests susceptibility in a few monocots against specific pathogens (Lu et al. 2006; Tian et al. 2014). In Arabidopsis, ethylene plays a role in dehydration-responsive stomatal closure (Daszkowska-Golec and Szarejko 2013), recovery during post-anaerobiosis reoxygenation (Tsai et al. 2014), and response to flooding and low oxygen (Bailey-Serres et al. 2012).

Ethylene has been shown to play a role in positive and negative regulation in the biosynthesis of specific SMs in a spatio-temporal manner in various plant species. In recent years, a review (Baharudin and Osman 2023) and a book chapter (Bhardwaj et al. 2022) focussing partially on this aspect have been published. The present review aims to provide a better depth on this subject. Here, we present a brief note on general mechanisms known for ethylene perception and signaling in plants and its interaction with other hormones. We then discuss in detail about the ethylene-mediated regulatory mechanisms playing a role in the biosynthesis of various SMs, which are derived through phenylpropanoid pathway (PPP), methylerythritol phosphate (MEP) pathway, mevalonic acid (MVA) pathway or other pathways. We also tried to simulate our present level of understanding about the related mechanisms and identify the gaps that need to be filled for further knowledge development and human benefits.

## Ethylene biosynthesis, signaling and interaction with other hormones

Ethylene biosynthesis, the shortest known pathway (two-step) for any hormone, starts with the formation of S-adenosyl-l-methionine (SAM) from l-methionine by SAM synthase which is then converted to 1-aminocyclopropane-1-carboxylic acid (ACC) by ACC synthase (ACS; carbon-sulfur lyases family). Gaseous hormone ethylene is released by further oxidisation of ACC by ACC oxidase (ACO; 2-oxoglutarate-dependent dioxygenase (2-OGD) family; ferrous-dependent non-heme oxygenase). Although known to form a heterodimer, ACS retains its activity in monomeric form (Kim and Yang 1992). Contrarily, ACO activity requires strict dimerization (Zhang et al. 2004). *ACS* and *ACO* belong to a multigene family with 8-19 and 3-6 members in various plants, respectively. ACS activity depends on the conservation of Lys-273, Arg-407, Tyr-233, and phosphorylation sites at the C-terminus region, which reduces its degradation (Liu et al. 2021). Their activity requires CO_2_ as an essential coactivator (Houben and Van de Poel 2019). Although ACOs are maintained at a higher ratio than ACS in the cell, their life span is of few minutes due to rapid proteolysis via oxidation (Barlow et al. 1997). 2-aminoethoxyvinyl glycine (AVG) is the most effective competitive ethylene biosynthesis inhibitor, inhibiting ACC synthase activity and inferring ethylene-mediated responses in plants (Saltveit 2005). Conversely, ethylene treatment can be provided to plants in an enclosed chamber with the regulated gas flow (1-1000 ppm) or through chemical agents like 2-chloroethylphosphonic acid (trade name: ethephon or ethrel) that decompose to release ethylene within cells below pH 4.0.

**Fig. 1:**
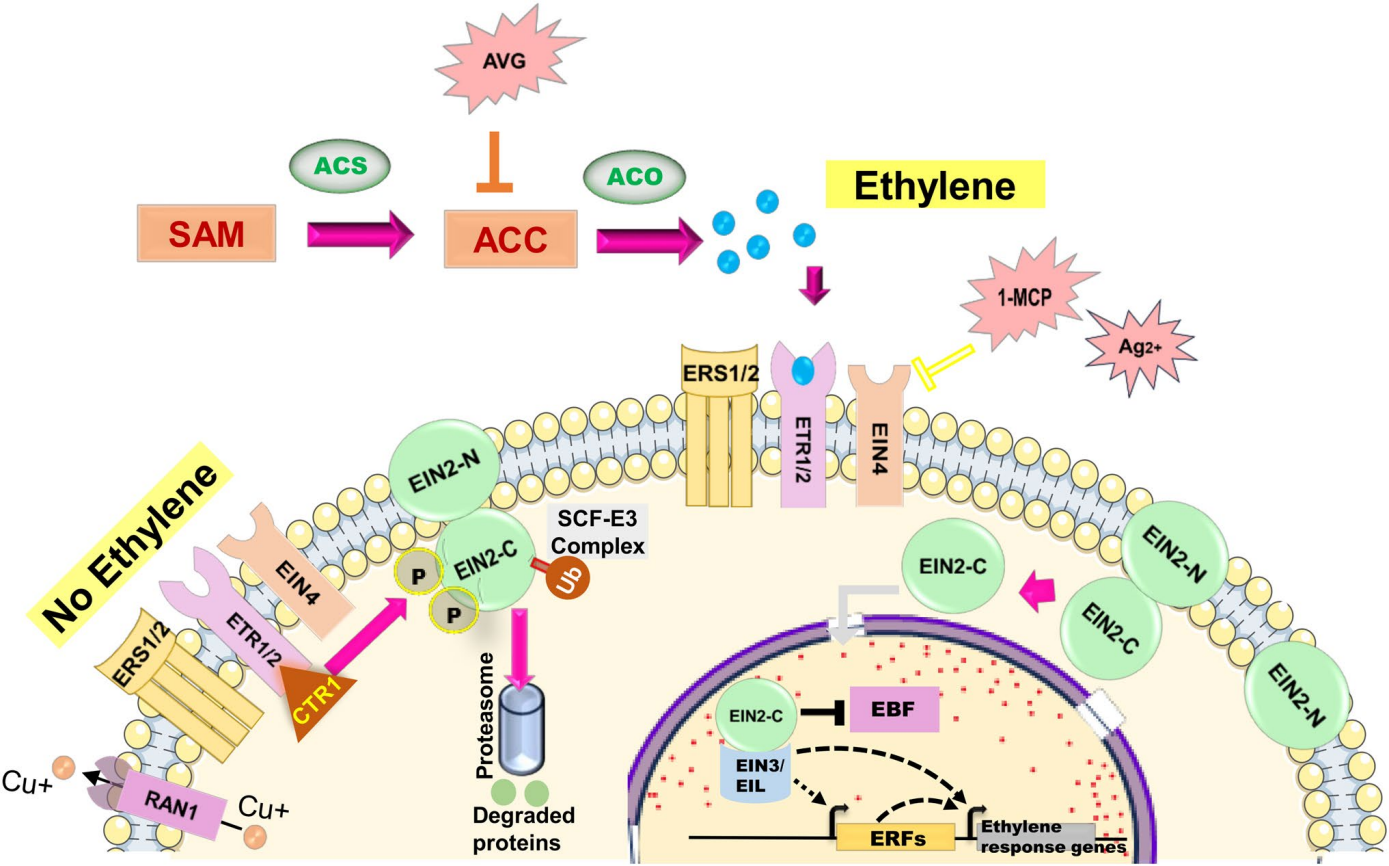
Diagrammatic representation showing ethylene biosynthesis and signaling in plants. Ethylene biosynthesis starts from SAM and is produced following the action of enzymes ACS and ACO. In the absence of ethylene, receptors (ETR)-CTR1 complex phosphorylates EIN2 leading to its proteasomal degradation via SCF-E3-mediaten ubiquitination (Ub). In presence of ethylene, CTR1 is inactivated and EIN2-C is released from EIN2-N by proteolytic cleavage to participate in transcription of downstream genes along with EIL. Ethylene, propylene and ethephon are commonly to treat plant for activating ethylene signaling. AVG, 1-MCP and Ag^2+^ are used as inhibitors of the pathway

Downstream ethylene receptor and signaling molecules have been characterised by epistatic analysis employing mutants like the *ethylene-insensitive 1* (*ein1*) or *ethylene-resistant 1* (*etr1*) of Arabidopsis, in which exogenous ethylene application failed to reverse the etiolated phenotype (Fluhr et al. 1996). While ethylene biosynthesis inhibitors were ineffective on mutants like *constitutive triple response 1* (*ctr1*), those like *ethylene-overproducer* (*eto*) showed inhibitor-depended reversal to wild-type phenotype (Stepanova and Ecker 2000). These analyses led to the identification of two ethylene receptors (ETR1/2) in Arabidopsis having three transmembrane α-helices for the ethylene binding ( Schott-Verdugo et al. 2019) and the ethylene response sensors (like ERS1, ERS2 and Ethylene Insensitive 4, EIN4) in Arabidopsis (Gao et al. 2008; Azoulay-Shemer et al. 2023). Similar to bacterial two-component systems, ETRs have histidine-kinase activity, marked by histidine autophosphorylation and phosphotransfer to aspartate residue that activates the pathway (Hung et al. 2016). Additionally, the GAF (cGMP-specific phosphodiesterases, Adenylyl cyclases, and FhlA)-domain mediates receptor heterodimerization, potentially for signal amplification and crosstalk with other receptors (Gao et al. 2008). CTR1 is the negative regulator of ethylene signalling. Its N-terminal domain interacts with ETRs and the C-terminus domain possesses Raf-kinases (Ser/Thr)-like activity (Lee and Yoon 2017). *EIN2*, *EIN3*, *EIN3-Like* (*EI*L), and the *ETHYLENE RESPONSE FACTORS* (*ERF*s; transcriptional regulators) are an absolute necessity for ethylene signaling (Lacey and Binder 2014). In the absence of ethylene, CTR1 possibly activates a MITOGEN-ACTIVATED PROTEIN KINASE KINASE (MAPKK), which phosphorylates EIN2 resulting in SCF-E3 (containing F-Box protein) mediated ubiquitination and proteasomal degradation (Wang et al. 2002). SCF-E3 also inactivates EIN3 and EIL1 (Guo and Ecker 2003). With ethylene binding, EIN2 phosphorylation is prevented either by loss of CTR1 kinase activity or its sequestration by the receptors (Binder 2020). The C-terminal end of EIN2 (EIN2-C) is separated from the N-terminal end (EIN2-N) by proteolytic activity, causing its translocation to the nucleus for transcriptional activities or sequestration in P-bodies, which otherwise remain in the endoplasmic reticulum (ER) (Merchante et al. 2015; Zhang et al. 2017). The EIN2-C binds to EIN3/EIL1 (downstream to EIN2) and is regulated by ubiquitination and proteasomal degradation by ethylene-binding F-box protein 1 (EBF1) and EBF2 (Binder et al. 2007). Homodimer of EIN3, EIL1, and EIL2 binds to the promoter region of *ERFs* (like *ERF1*), which in turn activates downstream genes by binding to GCC-box in the promoter (Hao et al. 1998) and modulates a range of physiological and developmental processes. The ethylene signaling cascade is conserved in monocots and dicots with minor differences. For example, in rice, the receptors are in continuous on-state even in the absence of ethylene (Adams-Phillips et al. 2004). 1-methylcyclopropene (1-MCP) and silver ions are known to inhibit ethylene signaling by forming a reversible receptor-inhibitor complex with ETRs and are employed for studying ethylene responses in plants(McDaniel and Binder 2012; Joy and Periyasamy 2020). A diagrammatic representation of ethylene biosynthesis and signaling is depicted in Fig. 1, and few key of the pathway in Arabidopsis genome are enlisted in Table 1.

**Table 1:** Genes of core ethylene pathway in *Arabidopsis thaliana*

S. no.	Gene	No. of copies in the genome	Function	Reference
1	*ACS*	12; one pseudogene (ACS3); two without catalytic activity (ACS10/12)	Synthesizes ACC from SAM; Rate-limiting step.	(Yamagami et al. 2003; McClellan and Chang 2008; Pattyn et al. 2021)
2	*ACO*	5	Oxidation of ACC to release ethylene, CO_2_ and cyanide	(Liu et al. 2021)
3	ETRs	2	Receptor for ethylene with histidine-kinase activity.	(Azoulay-Shemer et al. 2023)
4	Ethylene response sensor (ERS 1/2)	2	They have Serine/Threonine kinase activity. The transmembrane alpha helices form ethylene binding domain.	(Azoulay-Shemer et al. 2023)
6	Constitutive triple response (CTR1)	1	Negative regulator of ethylene, interferes with receptors	(Binder 2020)
7	Ethylene Insensitive 2/3 (EIN 2/3)	7	Regulates downstream signalling by binding to promoter region of ERFs	(Son et al. 2012)
8	Ethylene response factors (ERFs)	147	Binds primarily to GCC box of downstream genes and lead to transcriptional response	(Nakano et al. 2006)

As a plant hormone and signaling molecule, ethylene regulates several pathways that involve its interaction with other phytohormones or endogenous stimuli. These interactions are necessary to shape the developmental pathways and fine-tune various response mechanisms. The first mode of interaction between ethylene and other phytohormones involves influencing each other’s biosynthesis. For example, induction of ethylene biosynthesis by indole-3 acetic acid (IAA) treatment leads to root elongation in sugar beet (Khan et al. 2017). In Arabidopsis, the positive and antagonistic interaction between ethylene and jasmonic acid (JA) signaling involves the regulation of *LIPOXYGENASE* (*LOX*) genes (participates in JA biosynthesis) by EIN3 and EIL1 (Zhu et al. 2011; Song et al. 2014; Ma et al. 2020). Ethylene-induced JA biosynthesis has been found crucial for female flower development in *Cucurbita pepo* (Cebrián et al. 2023). In recent times, few climacteric fruit ripening has been found to occur through a Brassinosteroid (BR)-dependent induction of ethylene biosynthesis genes (Zhu et al. 2015; Guo et al. 2019). Similarly, strigolactone (SL) biosynthesis genes such as *MORE AXIALLY GROWTH 3* (*MAX3)* and *MAX4* are upregulated during ethylene-mediated leaf senescence (Ueda and Kusaba 2015). The second mode involves regulating the transport of other hormones by ethylene. For example, ethylene-mediated inhibition of root growth and cell elongation in Arabidopsis has been shown to mediate via induction of auxin transporters like *AUXIN INFLUX CARRIER* (*AUX1*) and *PIN-FORMED* (Rahman et al. 2001). The third mode involves influencing an important regulator or signal transducer, of other hormonal pathways. This makes the signaling network more complex and orchestrates several responses in plants. For example, auxin-induced AUX/IAAs are short-lived proteins that bind to AUXIN RESPONSIVE FACTORs (ARFs) and prevent transcriptional activation of genes. ACC treatment has been shown to increase the expression of *AUX/IAAs* without affecting the expression of *ARFs* in Arabidopsis (Harkey et al. 2018). Ethylene and gibberellins (GAs) are required for apical hook formation and light-induced hypocotyl elongation in Arabidopsis (Weiss and Ori 2007). In these cases, the influence of GA on ethylene biosynthesis and signaling is modulated by the interaction of DELLA repressors (like GIBBERELLIC ACID INSENSITIVE*,* GAI, and REPRESSOR of GAI*,* RGA) with ethylene response factors RELATED TO APETALA2.3 (RAP2.3) and RAP2.12 (la Rosa et al. 2014). In addition, induction of *GIBBERELLIN 2-OXIDASE6* (*GA2ox6*; by ethylene-responsive ERF5 and stabilization of DELLA repressors by ERF6 inhibits GA signaling under dehydration stress in Arabidopsis (Dubois et al. 2013). It has also been observed that SLs prevent hypocotyl elongation in light by stimulating *ELONGATED HYPOCOTYL5* (*HY5)* expression in an *MAX2*-dependent process (Jia et al. 2014). On the other hand, ethylene increases CONSTITUTIVE PHOTOMORPHOGENIS 1 (COP1)-mediated degradation of HY5 to assist hypocotyl elongation (Yu et al. 2013). Such interactions have been observed in a variety of plants, and their role in the biosynthesis of many SMs has been inferred, as discussed later.

### Role of ethylene in the regulation of metabolites derived from phenylpropanoid pathway

The role of ethylene is becoming increasingly clear in the regulation of biosynthesis of a large repertoire of SMs derived through PPP that involves aromatic amino acid phenylalanine as one of the precursors in dicots (Geng et al. 2020) and tyrosine in monocots (Barros et al. 2016). Based on the number of phenol rings and the structural elements binding the rings to one another, they are categorised as simple phenols, phenolic acids, flavonoids, xanthones, stilbenes, lignans, etc. (Vuolo et al. 2019). Phenolics have considerable structural diversity but are broadly classified into three classes: Class I—single benzene ring (C_6_ class), Class II—a C6 ring with four to seven attached carbon atoms (C_6_–C_n_ class), and Class III—a more complex skeleton in which second carbon is again attached to a benzene ring (C_6_–C_n_–C_6_ class) (Harborne 1989).

Anthocyanins (Class III—C_6_–C_2_–C_6_ phenolics) are well-known coloured water-soluble pigments which require enzymes DIHYDROFLAVANOL-4-REDUCTASE (DFR), ANTHOCYANIN SYNTHASE (ANS), and UDP-GLUCOSE/FLAVONOID 3-O-GLUCOSYLTRANSFERASE (UFGT) during later stages of biosynthesis and are valued for their antioxidant properties (Wang et al. 2022b). Their biosynthesis is regulated by a protein complex consisting of an R2–R3 MYB (subgroup, SG6 and SG4 majorly) transcription factor (TF), a basic-helix-loop-helix (bHLH) TF, and a WD-40 repeat protein (Lloyd et al. 2017; Naing and Kim 2018)**.** Although the involvement of ethylene in anthocyanin biosynthesis has been speculated since long through exogenous treatments, the underlying mechanisms are being uncovered by recent studies. In Arabidopsis, ethylene inhibits sucrose or light-mediated anthocyanin accumulation by the suppression of *PRODUCTION OF ANTHOCYANIN PIGMENT1*(*PAP1*; a R2–R3 *MYB* TF), *GLABRA3* (*GL3*, a *bHLH* TF), *TRANSPARENT TESTA8* (*TT8*; a *bHLH* TF) and sugar transporter *SUC1* along with overexpression of the negative regulator *MYBL2* (Jeong et al. 2010). Further, analysis of loss-of-function mutants revealed the role of *ETR1*, *EIN2* and *EIL2* in this signaling. In purple tomatoes, it was observed that fruits bagged at the immature stage but not at the red ripening stage could produce anthocyanin when unbagged (Xu et al. 2022).Further transcriptome analysis indicated upregulation of ethylene biosynthesis (*ACS2* and *ACO-*related) and signaling (*GRL2, NR/ETR3,* and *EIL3*) genes along with a few *AP2/ERF* and *R2-R3 MYBs* in the red ripening stage compare to immature fruits. Ethylene treatment caused suppression of structural (*F3H*, *F3′5′H*, *DFR* and *ANS*) and regulatory genes (*SlAN1* and *SlAN2-*like) involved in anthocyanin biosynthesis. Contrarily, in many fruits like plums, strawberries, grapes, and mango (fruit peel), role of ethylene in promoting anthocyanin has been revealed through exogenous treatments, correlation studies between anthocyanin biosynthesis and expression of ethylene pathway genes, and VIGS analyses (El-Kereamy et al. 2003; Lopes et al. 2015; Farcuh et al. 2022; Xiao et al. 2022). Other environmental signals like light and temperature, and endogenous signaling molecules like melatonin have been shown to influence ethylene-induced anthocyanin accumulation in fruits like grapes and tomatoes (El-Kereamy et al. 2003). In apples (a climacteric fruit), anthocyanin biosynthesis (fruit colouration) coincides with the release of ethylene during fruit ripening and binding of ethylene-induced MdEIL1 to *MdMYB1* (an SG6, R2–R3 type, positive regulator for anthocyanin biosynthesis) promoter was observed. MdEIL1 in turn, directly activates the *ERF3* gene and participates in a positive feedback loop (Wang et al. 2022a). In a more recent study, an ethylene-induced *MdMYB17* (an SG4 R2–R3 type) was established as a negative regulator of anthocyanin biosynthesis that mediates its action by binding to *MdMYB1* and *MdEIL1* promoters (Wang et al. 2022a). Interestingly, an interaction between MdEIL1 and MdMYB17 suppresses this negative regulation, suggesting a calibration of anthocyanin biosynthesis in apple fruit by ethylene through a regulatory module involving MdMYB1, MdMYB17 and MdEIL1. These studies indicate specificities acquired in ethylene signaling during the evolution of various plant species to regulate widely found metabolites like anthocyanin that might aid them in their adaptability to the environment.

Flavonols (Flavonoids with ketone ring; Class III C_6_–C_3_–C_6_ phenolics) are antioxidants that take part in plant response to external stimuli and developmental cues. Recent research indicates that ethylene, in association with other hormones or signaling molecules regulates the level of flavonols in various plant parts and helps maintain a threshold ROS level in plants. Mutants (like *eto1*) analyses and exogenous treatments have shown a role for ethylene (along with auxin) in the accumulation of flavonols and its derivatives (like quercetin and kaempferol) in Arabidopsis roots, which itself regulate auxin transport and related physiological responses (Lewis et al. 2011). Increased flavonol biosynthesis accompanied by strong expression of flavonoid pathway gene *CHALCONE SYNTHASE* (*CHS*) occurs in guard cells of wild-type Arabidopsis (Col-0) but not in *transparent testa 4-2* (*tt4-2*; null mutation in *CHS*) plants (Watkins et al. 2014). The mutant, devoid of the antioxidant activity of flavonols, shows ABA hypersensitivity for stomatal closure due to ROS burst. Similarly, increased levels of quercetin and kaempferol,and decreased levels of ROS in wild-type plants led to modulated stomatal closure in response to ethylene treatment, a phenomenon that was not observed in *ein2* mutants (Azoulay-Shemer et al. 2023). These observations suggest a clear role of ethylene signaling in flavonol biosynthesis in guard cells. More recently, NtMYB184 (flavonol-specific, SG7 R2–R3 type) has been shown to activate many flavonol biosynthesis genes in tobacco. The silencing (RNAi) and knockout (genome editing) of this gene led to a depletion of flavonols in the guard cells (Song et al. 2023). *NtMYB184* expression is repressed and stimulated by ABA and ethylene, respectively. Additionally, the ability of ethylene to suppress ABA-mediated flavonol depletion, ROS accumulation and stomatal closure is lost in the absence of *NtMYB184* function. Thusestablishing it as an indispensable link between ABA and ethylene signaling in the tobacco guard cells. Ethylene also influences flavonoid accumulation in various fruits. In Chinese red pear fruits, 1-MCP induced red colouration (Ni et al. 2020). It was further observed that methyl jasmonate (MJ) alone could induce yellow colouration due to the accumulation of flavonoids other than anthocyanin which is mediated through the induction of ethylene biosynthesis genes (like *PpACS*, *PpACO*, *PpEIN3/EIL*, *PpERF1*) and could not be achieved in the presence of 1-MCP. The activated ethylene signaling suppresses the activity of the MYB-bHLH-WD-40 complex by downregulating *PpMYB10* and *PpMYB14*, which consequently inhibits anthocyanin biosynthesis. Thus, it appears ethylene may participate in reorienting jasmonate-mediated flavonoid biosynthesis in Chinese red pear by suppressing anthocyanin biosynthesis which drives the pathway towards biosynthesis of other important flavonoids.

Isoflavones are plant phenolics, found predominately in Fabaceae, that are highly valued as pharmacological agents for having phytoestrogen-like (17-β-estradiol-like) activity (Wang et al. 2022c). Functioning as attractants released by plants, they help maintain a symbiotic relationship with rhizospheric bacteria (Liu et al. 2007). Ethephon treatment causes high accumulation of isoflavones (like daidzein and genistein) in soybean sprouts and postharvest leaves. This results from increased expression of related biosynthetic genes including *ISOFLAVANOL SYNTHASE* (*IFS*), *ISOFLAVONE 7-O-URIDINE DIPHOSPHATE GLYCOSYLTRANSFERASE* (*IF7GT*) and *ISOFLAVONE7-O-GLUCOSIDE-6-O-MALONYLTRANSFERASE* (*IF7MαT*) (Yuk et al. 2016; Yin et al. 2023). In soybeans, ethylene also mediates serotonin and melatonin-dependent isoflavone accumulation under temperature stress (Kumar et al. 2021). In *Medicago truncatula*, many isoflavones (like liquiritigenin, formononetin, medicarpin, and biochanin A) act as phytoalexins and hold a centre stage in plants’ resistance to *Rhizoctonia solani* which is compromised in *sikle* (*skl*) plants, a mutant defective for ethylene biosynthesis (Liu et al. 2017; Kidd et al. 2021). These reports indicate that ethylene-dependent maintenance of specific isoflavone levels is crucial for plant development and stress response in members of Fabaceae. Treatment with ethephon and 1-MCP also indicated the involvement of ethylene in the regulation of specialized metabolites like capsaicinoids, a group of 22 phenylpropanoid derivatives dominated by capsaicin that are found only in the placental tissue of *Capsicum* fruits (Wen et al. 2022). Thus, it appears that ethylene signaling has evolved to regulate the biosynthesis of phenolic compounds present in specific groups of plants. However, more work needs to be done to identify the precise regulatory mechanism in these cases.

Reports during the past few years also indicate the role of ethylene alone or in association with various regulators in the biosynthesis of other widely distributed plant phenolics. For example, hydroxycinnamic acid amides (HCAA; polymer of hydroxycinnamic acid and polyamines, Class II—C_6_C_3_ phenolics) are important for plant development and immunity (Bassard et al. 2010)**.** AGMATINE COUMAROYL TRANSFERASE (ACT) is an important enzyme for the biosynthesis of HCAA like *p*-coumaroylagmatine (CouAgm). *OCTADECANOID-RESPONSIVE ARABIDOPSIS 59* (*ORA59*), encoding a key AP2/ERF TF for JA and ethylene signaling, was shown to get synergistically induced by ethylene and JA, and directly activates the *AtACT* gene (Li et al. 2018)*.* This suggests a role for *ORA59*-mediated JA/ethylene signaling regulating of HCAA levels in Arabidopsi*s*. Scopoletin, a coumarin compoundis valued both as phytoalexin and therapeutic. In tobacco, it is induced by JA signaling in response to the fungal pathogen *Alternaria alternata.* It has been revealed that JA mediates this action by inducing ethylene biosynthesis genes, and mutants compromised for ethylene signaling (*irACO* and *Ov-etr1*) have reduced levels of scopoletin (Jia et al. 2013). More recently, receptor kinase NaLRR-RK4 and TF NaERF109 have been found to participate in pathogen-induced scopoletin biosynthesis by regulating the expression of *DEFENCIN 19 (NaDEF19)* and *NaF6’H* (Zhao et al. 2022). Exogenous ethephon treatment significantly induced scopoletin in *Morinda citrifolia* fruits and suspension culture (Jia et al. 2020). Thus, the ethylene pathway positively regulates the biosynthesis of phenolics like scopoletin and HCAA across species which have uniform roles in plant defence mechanisms.

Lignin, a component of the plant cell wall, belongs to a class of polyphenols with a structure composed of monolignol and lignan units polymerized into a three-dimensional network (Liu et al. 2018). In *Vigna radiata,* ethylene stimulates lateral root formation and lignification with enhanced activity of α-EXPANSINS and XYLOGLUCAN ENDOTRANSGLUCOSYLASES/HYDROLASES (XTH) (Huang et al. 2013). Exogenous application of ethephon and overexpression of *ERF1b*-like gene in pear fruits result in higher lignification accompanied by increased expression of lignin biosynthesis genes (Jin et al. 2022). In the same way, ethephon and 1-MCP treatment increases and decreases lignin content in kiwi fruits, respectively, and accordingly affects many phenylpropanoid pathway genes like *PAL, C4H and CHS* (Choi et al. 2023). However, in Arabidopsis, ethylene has been shown to negatively regulate the lignification during the formation of leaf serration through *EIN*3, which upregulates laccase targeting *miR397b* (Gaddam et al. 2022). The ethylene may be involved in fine-tuning structural components of plant cells by regulating the biosynthesis of SMs like lignin.

**Fig. 2:**
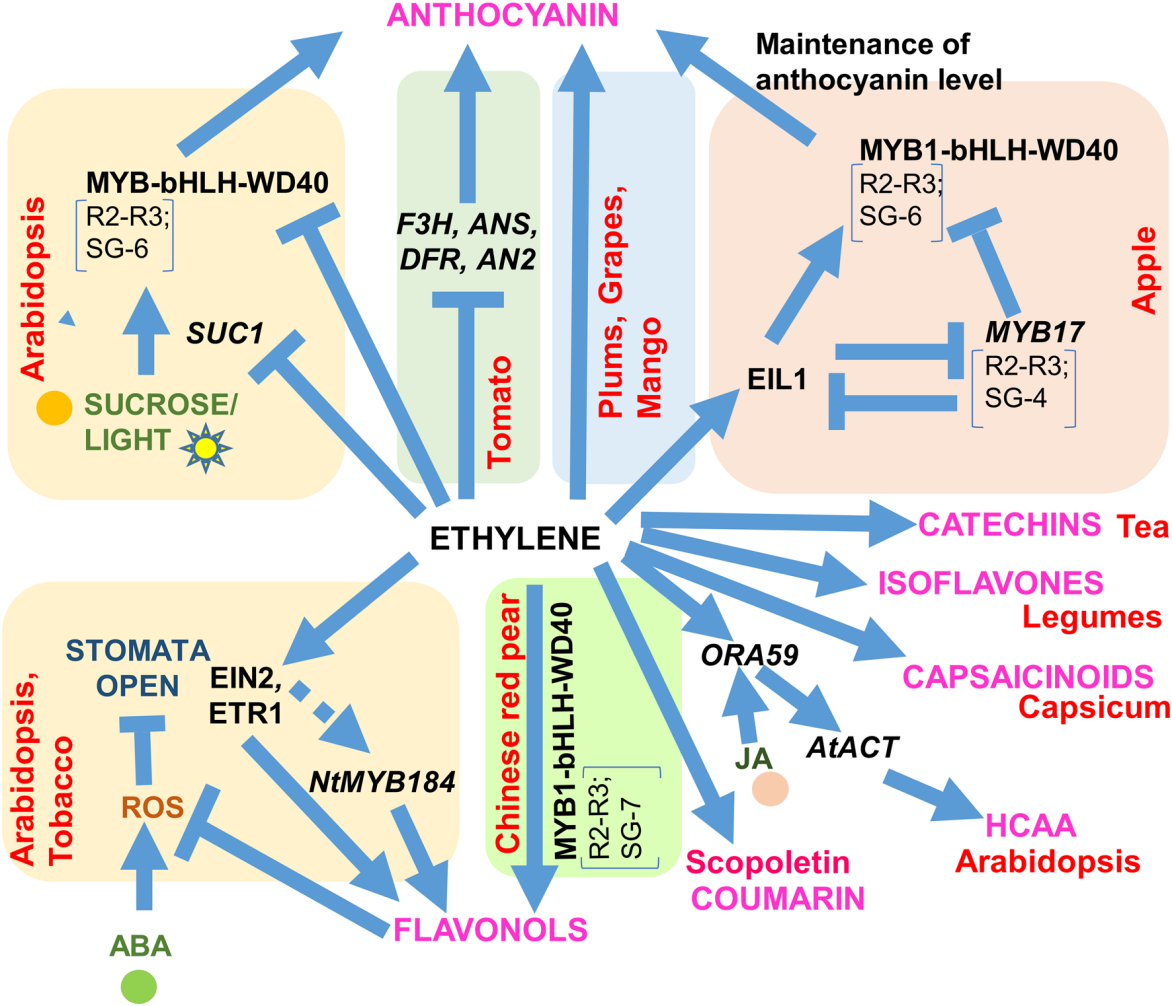
Schematic representation showing effect of ethylene on biosynthesis of SMs that are derived through PPP. The classes and/or names of SMs (case-specific manner) are indicated along with the plant name. The related gene/protein, and the regulators or regulator complexes are also shown. Role of other signaling molecule like JA, light, sucrose or ABA which interacts with ethylene pathway are also mentioned.

Resveratrol, obtained from grapes, peanuts and *Polygonum,* is used as an antiplatelet agglutination, anticancer, antioxidant, and has antiviral activities. Resveratrol content reportedly increased linearly in peanut seedlings treated with varying concentrations of ethylene and copper ion, a cofactor for ethylene receptor (Bao et al. 2021). Likewise**,** treatment of oolong tea leaves with ACC escalated total catechin content, including epigallocatechins (EGC), epigallocatechin-gallate (EGCG), and epicatechin (EC), and other phenolics leading to enhanced flavour of tea (Ke et al. 2018). However, in all these cases, the mechanism for ethylene-mediated regulation remains to be delivered. A schematic representation of all the PPP-derived SMs influenced by ethylene along with their regulatory factors, is given in Fig. 2.

### Role of ethylene in the regulation of metabolites derived from MEP/MVA pathway

The mevalonic acid (MVA) pathway starts from the condensation of two molecules of acetyl-CoA to generate isopentenyl pyrophosphate (IPP) and dimethylallyl pyrophosphate (DMAPP) through a series of condensation, isomerisation, phosphorylation, and decarboxylation steps. Similarly, the methylerythritol phosphate (MEP) pathway (in chloroplast) also generates IPP and DMAPP, but with different precursors. Both MEP and MVA pathways converge and lead to the synthesis of various compounds related terpenes and sterols (Zhao et al. 2013; Chatzivasileiou et al. 2019). Sesquiterpenes are a C-15 class of terpenoids consisting of three isoprene subunits (Asai et al. 2020; Xu et al. 2023)**.** Rubber from *Hevea brasiliensis* is an economically important sesquiterpene-polymer. Ethephon stimulation has become a common practice for rubber plantations worldwide. Ethylene enhances the latex yield by the rapid acceleration of the glycolysis, which in turn replenishes the precursors like acetyl-CoA that are required for the biosynthesis of IPP and natural rubber molecules (Chow et al. 2012; Liu et al. 2016). It also regulates the water flow between latex cells and inner liber by increasing cell permeability through two aquaporins genes, *TONOPLAST INTRINSIC PROTEIN1* (*HbTIP1*) and *PLASMA-MEMBRANE INTRINSIC PROTEIN*2 (*HbPIP2*), and leads to prolonged latex flow (Tungngoen et al. 2009; d’Auzac et al. 2018). A role for ethylene in valencene (valuable sesquiterpenoid) biosynthesis by CitAP2.10 (AP2/ERF)–mediated upregulation of *TERPENE SYNTHASE 1* (*CsTPS1*) has been reported in sweet orange (Shen et al. 2016). Ethylene-mediated increase in sesquiterpenoids has also been reported in the Chinese medicinal plant *Atractylodes lancea* (Yuan et al. 2016).

Carotenoids are 40-carbon isoprenoids with polyene chains and play an essential role in photosynthetic protection mechanisms. Their biosynthesis utilizes GGPP from the MEP pathway and starts from phytoene, which leads to the synthesis of various types of carotenes (Park et al. 2002; Yuan et al. 2022) and for its biosynthesis. Regulation of *PHYTOENE SYNTHASE (PSY),* the key rate-limiting enzyme, by ethylene has been known for long (Maunders et al. 1987), but knowledge about the regulatory mechanism remained elusive. However, a study in Citrus indicates that, ERF061 alone could regulate nine steps of the carotenoid biosynthesis by activating the related genes, including *PSY1* (Zhu et al. 2021). Similarly, ethylene treatments increased carotenoids content in tomatoes (Sun et al. 2020). However, RNAi-mediated silencing of ethylene-induced *SlERF6* in tomatoes resulted in elevated content of carotenoids with higher expression of *1-DEOXY-D-XYLULOSE-5-PHOSPHATE (DXS)* and *HEAT SHOCK PROTEIN 21*(*HSP21*) genes (Lee et al. 2012;)., These studies suggests complexities in ethylene-mediated regulation of carotenoid biosynthesis.

Terpenoid indole alkaloids (TIA) are high-value therapeutic SMs found in Apocynaceae and Rubiaceae family. They are biosynthesized by the conjugation of indole alkaloids to isoprene units. Vinblastine (a TIA) from *Catharanthus roseus* is highly valued for antineoplastic activity. Ethephon-treated *C. roseus* seedlings have two-fold more vinblastine content than control due to increased accumulation of *DESACETOXYVINDOLINE-4-HYDROXYLASE* (*D4H*), *TABERSONINE-16-HYDROXYLASE* (*T16H*) and *DEACETYLVINDOLINE-4-O-ACETYLTRANSFERASE* (*DAT*) which are critical downstream genes of TIA pathway (Wang et al. 2016). In the *C. roseus* cell suspension cultures*,* ajmalicine (a monoterpenoid indole alkaloid) biosynthesis was reportedly enhanced by the combined application of ethephon and cytokinin. This correlates to the elevated expression level of *GERANIOL-10-HYDROXYLASE* (*G10H*) gene (Papon et al. 2005). Although a synergistic effect of ethylene (enhances MVA pathway) and methyl jasmonate (enhances MEP pathway) on TIA content has been reported in *Catharanthus roseus*, ethylene alone causes hyper-accumulation of nine metabolites including ajmalicine, tabersonine, and catharanthine (Zhang et al. 2018). A decrease in primary metabolites was also observed, which might have directed the precursors towards the TIA pathway. Ganodermic acid (lanosterol-derived triterpenoid) from *Ganoderma* *lucidum* has important pharmacological properties. It is enhanced by short-duration ethylene treatment, which due to the upregulation of *HYDROXYMETHYLGLUTARYL-COA SYNTHASE* (*HMGS),* *SQUALENE SYNTHASE* (*SQS), ACAT,* and *MEVANOLATE KINASE* (*MVK)* along with genes for amino acid and glucose metabolism (Meng et al. 2022).

**Fig. 3:**
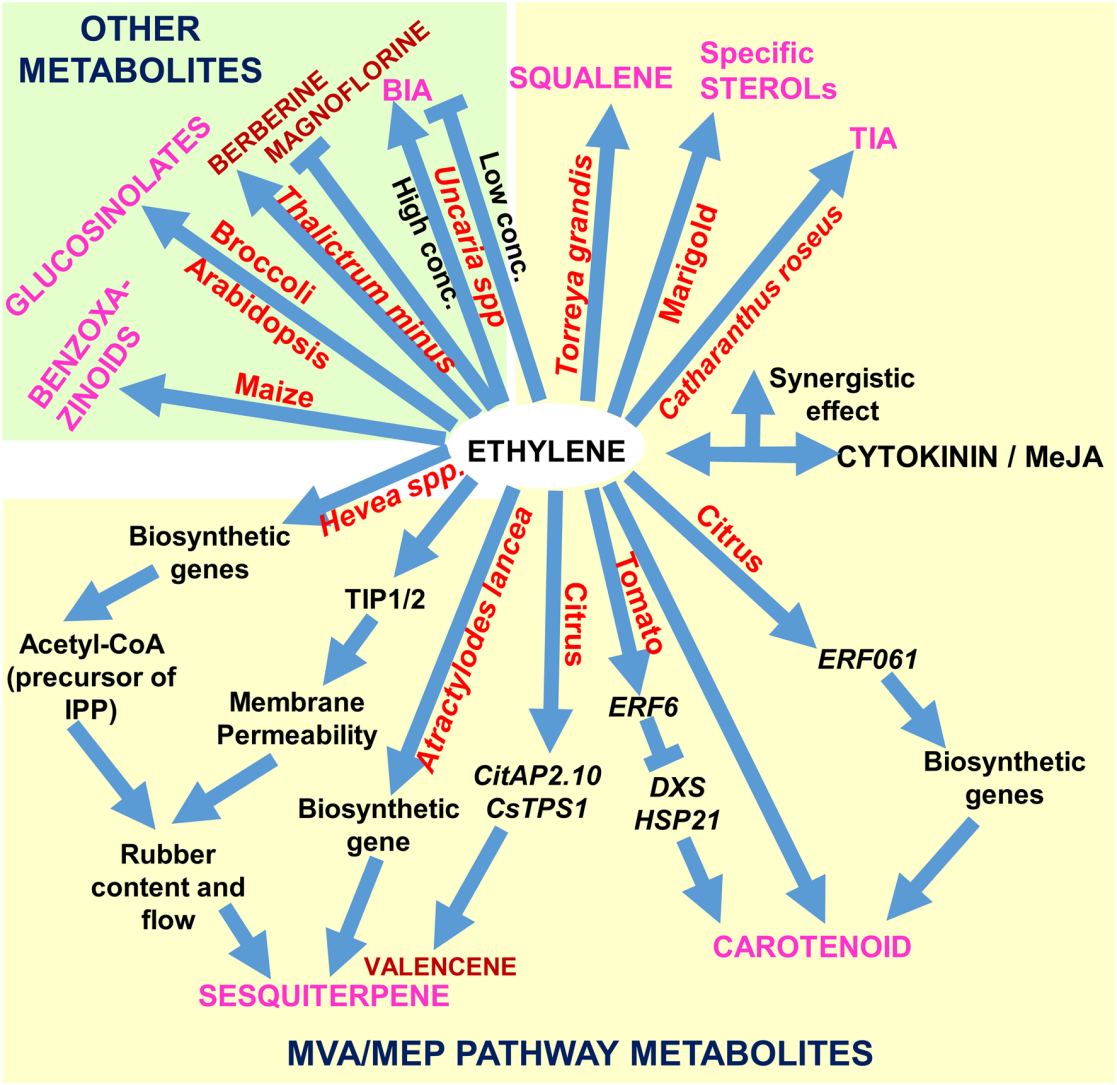
Schematic representation showing the influence of ethylene on secondary metabolites derived through MEP/MVA or other pathways (neither PPP nor MVA/MEP). The classes and/or names of SMs are indicated along with the plant name. The related pathway genes or proteins are also shown.

Phytosterols are also isoprenoid compounds synthesized via the MVA/MEP pathway that are parts of the membrane system with a role in growth, regulation, and signaling (Bootter et al. 2023). Post-harvest ethylene application on *Torreya grandis* nuts results in a strong increase in squalene (an intermediate of the terpene-biosynthetic pathway) and soyasapogenol B, along with a slight negative impact on the accumulation of β-sitosterol (Hu et al. 2022). Concurrently, the transcripts of *GERANYLGERANYL PYROPHOSPHATE SYNTHASE* (*TgGGPS)* were upregulated indicating the suitability of an ethylene-releasing agent for post-harvest treatment of the nuts. Esculeoside A (a steroidal glycoalkaloid) accumulation in tomatoes depends on ethylene signaling. Ethylene plays a critical role in one of the glycosylation steps, as confirmed in *rin* and *nor* mutant lines that fail to ripe due to ethylene deficiency (Iijima et al. 2009). An interesting analysis done in the hairy root culture of *Calendula officinalis* indicates a major role of ABA in triterpenoid biosynthesis. In contrast, ethylene was shown to have a profound effect on sterol metabolism, specifically regulating the ratio between stigmasterol to sitosterol (Markowski et al. 2022). From the aforementioned reports, it may be inferred that ethylene has a significant role in the regulation of metabolites biosynthesized through MVA/MEP pathways; however, the mechanism has been poorly studied and understood. Most of these terpenes or sterols have high commercial importance and, hence, have been studied mainly in non-model plants. However, with the advent of genomic and molecular tools, the observed mechanism is expected to be uncovered soon. A schematic representation of all the MEP/MVA pathway-derived SMs influenced by ethylene is given in Fig. 3.

### Ethylene-mediated regulation of secondary metabolites biosynthesized through other pathways

In the above two sections, ethylene-mediated regulation of SMs derived from PPP and MVA/MEP pathways was discussed. However, the role of ethylene in regulating the abundance of a few SMs that are synthesized without the participation of these pathways has also been studied. Glucosinolates, benzylisoquinoline alkaloids and benzoxazinoids are such specialized metabolites which are derived from various amino acids. Glucosinolates are prevalent in members of family Brassicaceae. They are derived from different amino acids and are accordingly grouped as aliphatic, aromatic or indole glucosinolates (Kitainda and Jez 2021).In Broccoli, using diphenyleneiodonium (DPI, a ROS inhibitor) and 1-MCP, it was estimated that ROS critically contributes to the accumulation of all major indole glucosinolates under wounding stress, whereas ethylene participates in the induction of specific indole glucosinolates such as neoglucobrassicin and 4-hydroxy-glucobrassin (Torres-Contreras et al. 2018). In Arabidopsis roots, specific downregulation of few glucosinolates like 4-methylsulfinylbutyl glucosinolates, 7-methylsulfinylheptyl glucosinolates, 8-methylsulfinyloctyl glucosinolate, 4-methylthiobutyl glucosinolate, 5-methylthiopentyl glucosinolate, and hydroxy-8-methylsulfinyl octylglucosinolates was observed under ACC treatment by complete metabolome analysis (Hildreth et al. 2020). Participation of JA/ethylene signaling in *ORA59*-mediated induction of aliphatic glucosinolates in response to Rhizobacterial colonization in Arabidopsis has also been documented (Pangesti et al. 2016)**.** Benzoxazinoids are allelopathic and defence response-related specialized metabolites with predominance in the family Poaceae. They are biosynthesized from tryptophan through indole formation. In maize, biosynthesis of the anti-insect benzoxazinoids like 2,4-dihydroxy-7-methoxy-1,4-benzoxazin-3-one (DIMBOA) and DIMBOA-glucoside (DIMBOA-Glc) were shown to be regulated by a MYB TF and ethylene-induced *ZmMPK6* which function downstream of ethylene signaling (Zhang et al. 2021). The Benzylisoquinoline alkaloids include medicinally important ~2500 diverse structures that are biosynthesized from tyrosine via S-reticuline. In *Thalictrum minus* cell suspension cultures, ethephon treatment increased berberine (a BIA) biosynthesis through increased activity of TETRAHYDROBERBERINE (THB) OXIDASE, whereas magnoflorine (another BIA) was induced by inhibiting ethylene biosynthesis using silver ions or AVG (Kobayashi et al. 1991). More recently*, *the application of ethephon at 36mM concentration decreased the rhynchophylline and isorhynchophylline (both BIAs) content in *Uncaria,* while their increased accumulation was observed at 18mM concentration. This alteration in metabolite content also correlates to the expression level of the biosynthetic enzymes and transcriptional regulators including positive regulators, such as *bHLH1*, *bHLH2* and *ERF1*, and negative regulators like *NAC1* (Li et al. 2022). Among other metabolites, aromatic aldehydes like octanal, decanal, nonanal and alcohols like nonanol were markedly enhanced by ethylene treatment and improved the quality of the ripened papaya fruits (Der Agopian et al. 2020). The aforementioned instances indicate that ethylene influence in various secondary metabolites biosynthesis is limited to specific pathways rather than having a global effect on larger classes of metabolites. However, the regulation of these pathways under ethylene signalling needs to be studied further in detail.

### Complexities in ethylene biosynthesis and probable implications on secondary metabolism

ACC is long known to form various conjugates like 1-malonyl-ACC (MACC), γ-glutamyl-ACC (GACC), jasmonyl-ACC (JA-ACC) which are catalysed by ACC N-malonyl transferase (AMT), JA-ACC by JA amino acid synthase (JAR1) and GACC by γ-glutamyl-transferase (GGT), respectively, in plants (Amrhein et al. 1981; Martin et al. 1995; Staswick and Tiryaki 2004). However, the specific role of these conjugates remains to be discovered. It is believed they are transported over long distance and across the plasma membrane by ACC transporter protein (*LYSINE HISTIDINE TRANSPORTER 1, LHT1*) (Shin et al. 2015). Ethylene reportedly stimulates the formation of MACC (Liu et al. 1985; Pettigrew 2008). It is believed that these conjugates maintain a pool of ACC which is broken down to release ethylene under demanding conditions. Interestingly, few instances have been observed that indicate the role of ACC and ACC conjugates in alternate pathways. For example, Arabidopsis *fei1 fei2* has reduced cellulose microfibrils in the cell wall at the root tip which was reversed by application of ethylene biosynthetic inhibitor (targeting ACS) but not by signaling inhibitor (Xu et al. 2008). Similarly, ethylene biosynthesis and not the signaling inhibitor could reverse isoxaben (a cellulose biosynthesis inhibitor)-induced inhibition of root cell expansion (Tsang et al. 2011). An Arabidopsis octuple *ACS* mutant displays embryo lethality but the same has not been observed for a mutant having a defect in ethylene signaling (Mou et al. 2020). More recently, dual activity for a few ACS proteins with an additional involvement in pyruvate biosynthesis has also been reported (Xu et al. 2021). All these reports suggest a probable specific role of *ACS* and ACC in plant systems outside the ethylene signaling pathway. It cannot thus be ignored that *ACS* and ACC may regulate secondary metabolism from an alternative pathway. However, further detailed research is required.

## Conclusion

Ethylene is associated with the regulation of diverse groups of SMs synthesized through PPP, MVA/MEP, and other pathways. Studies done in recent times indicate that ethylene signaling influences the central regulatory domain of a specific class of metabolites, like the MYB-bHLH-WD40 which is involved in SM biosynthesis through PPP. However, with evolution, the pathway is specialized to impart a negative or positive impact on the biosynthesis of the same or similar metabolites across species, organs, tissues and conditions. In general, ethylene, possibly in association with other hormones, fine-tunes the biosynthesis of SMs. In some cases, it strictly favours the synthesis of a few members of a certain class of compounds as observed in the case of sterols and their derivatives. SMs are proving to be the ultimate effectors of plant response to developmental signals and external stimuli. They are equally useful for mankind as medicinal, nutraceutical and aromatic resources. However, most of them are synthesized at a low level and, the knowledge about the whole biosynthesis pathway is limited. Therefore, it becomes more important to gain insight into the regulatory pathways which could be used to engineer better and more useful varieties with better yield, adaptability and produce the desired SMs in quantities enough to fulfill mankind’s requirements. Ethylene signaling could be utilized to bring specific changes in the plant and the SM pool. However, more studies are required on the pathways associated with larger groups of plant species, which contribute to the diversity of compounds produced by the plant kingdom, especially those related to the MVA/MEP pathway and other processes.
